# Maternal depression, antidepressant prescriptions, and congenital anomaly risk in offspring: a population-based cohort study

**DOI:** 10.1111/1471-0528.12682

**Published:** 2014-03-11

**Authors:** L Ban, JE Gibson, J West, L Fiaschi, R Sokal, L Smeeth, P Doyle, RB Hubbard, LJ Tata

**Affiliations:** a Division of Epidemiology & Public Health, University of NottinghamNottingham, UK; b Nottingham Digestive Diseases Centre, National Institute for Health Research Biomedical Research Unit, Nottingham University Hospitals National Health Service TrustNottingham, UK; c Department of Non-communicable Disease Epidemiology, London School of Hygiene & Tropical MedicineLondon, UK

**Keywords:** Antidepressants, congenital anomaly, depression, SSRIs, TCAs

## Abstract

**Objective:**

To estimate risks of major congenital anomaly (MCA) among children of mothers prescribed antidepressants during early pregnancy or diagnosed with depression but without antidepressant prescriptions.

**Design:**

Population-based cohort study.

**Setting:**

Linked UK maternal–child primary care records.

**Population:**

A total of 349 127 singletons liveborn between 1990 and 2009.

**Methods:**

Odds ratios adjusted for maternal sociodemographics and comorbidities (aORs) were calculated for MCAs, comparing women with first-trimester selective serotonin reuptake inhibitors (SSRIs) or tricyclic antidepressants (TCAs) and women with diagnosed but unmedicated depression, or women without diagnosed depression.

**Main outcome measures:**

Fourteen system-specific MCA groups classified according to the European Surveillance of Congenital Anomalies and five specific heart anomaly groups.

**Results:**

Absolute risks of MCA were 2.7% (95% confidence interval, 95% CI, 2.6–2.8%) in children of mothers without diagnosed depression, 2.8% (95% CI 2.5–3.2%) in children of mothers with unmedicated depression, and 2.7% (95% CI 2.2–3.2%) and 3.1% (95% CI 2.2–4.1%) in children of mothers with SSRIs or TCAs, respectively. Compared with women without depression, MCA overall was not associated with unmedicated depression (aOR 1.07, 95% CI 0.96–1.18), SSRIs (aOR 1.01, 95% CI 0.88–1.17), or TCAs (aOR 1.09, 95% CI 0.87–1.38). Paroxetine was associated with increased heart anomalies (absolute risk 1.4% in the exposed group compared with 0.8% in women without depression; aOR 1.78, 95% CI 1.09–2.88), which decreased marginally when compared with women with diagnosed but unmedicated depression (aOR 1.67, 95% CI 1.00–2.80).

**Conclusions:**

Overall MCA risk did not increase with maternal depression or with antidepressant prescriptions. Paroxetine was associated with increases of heart anomalies, although this could represent a chance finding from a large number of comparisons undertaken.

## Introduction

Antenatal depression is estimated to affect 8–11% of women in high-income countries,^1^ and the proportion of pregnant women prescribed antidepressants has increased dramatically in the last two decades.^2^ Whereas it is important to manage antenatal depression, as it may confer harmful effects if left untreated,^3^,^4^ there is conflicting evidence for the safety of antidepressant use during early pregnancy, particularly for congenital anomaly risks.^5^,^6^ Following drug company warnings in 2005 about paroxetine-associated cardiac malformations, based on extremely limited evidence,^7^ there was an increase in studies assessing the potential teratogenicity of antidepressants, with almost all focusing on SSRIs.^8^–^31^ The results, mostly from European and North American countries, have been extremely mixed,^6^ with some showing similar excess congenital anomaly risks for other SSRIs, including fluoxetine, sertraline, and citalopram,^14^,^19^,^22^,^24^,^26^,^27^,^29^ and evidence of no increased risks with paroxetine.

Despite these inconsistencies, specific warnings against paroxetine use in pregnancy have been incorporated into some current national guidelines,^32^–^34^ which may inappropriately infer the relative safety of other SSRIs and antidepressant classes, and disqualify the potential risk contribution of women's underlying mental illness and physical health. In fact, few studies have been large enough to assess risks of specific anatomical subgroups with individual SSRIs. Despite the continued use of TCAs by a significant proportion of pregnant women,^35^ estimates of the relative harm of TCAs are also lacking, with only five published studies reporting results for some selected system-specific anomalies.^12^,^25^,^29^,^30^,^36^ A Danish study assessed potential confounding by underlying depression by comparing women exposed to SSRIs with women who paused their use of SSRIs during pregnancy;^29^ however, no studies have assessed diagnosed but unmedicated depression, which accounts for a much larger proportion of women potentially at risk.^4^,^35^

Despite many women discontinuing antidepressants in pregnancy,^37^ the UK has one of the highest proportions of pregnant women internationally being prescribed antidepressants, yet there remain no population-based studies assessing the teratogenicity of individual antidepressant drugs. To inform treatment guidelines in pregnancy, we conducted a cohort study using primary care data from a representative national UK population to: (1) provide estimates of absolute and relative risks of congenital anomaly and system-specific anomalies in children born to women without depression, with unmedicated depression during the first trimester of pregnancy, and with SSRIs or TCAs in the first trimester; (2) estimate drug class-related risks for individual heart anomalies; and (3) estimate system-specific congenital anomaly risks for individual SSRIs.

## Methods

### Study population

We studied all singleton live births for women aged 15–45 years between 1990 and 2009 from The Health Improvement Network (THIN), in which the medical records of the mothers and the children were linked to provide prospectively recorded information throughout pregnancy and in the year before pregnancy. THIN is a nationally representative database of computerised primary care records from across the UK that has been validated for pharmacoepidemiology studies, and contains diagnoses, events, symptoms, and drug prescriptions.^38^ We excluded 9096 children (2.5% of the study population) whose mothers had bipolar disorder, schizophrenia, other serious psychotic disorders, or prescriptions for antimanic and antipsychotic drugs before childbirth.

### Defining all major and system-specific congenital anomalies

All diagnoses of major congenital anomalies (MCAs) were identified in the children's medical records using Read codes that we classified into 14 system-specific groups according to the European Surveillance of Congenital Anomalies (EUROCAT) subgroups,^39^ which are based on the codes listed in the tenth edition of the International Classification of Diseases (ICD–10, mainly in chapter Q). A comparison of prevalence estimates across all system-specific groups (and specific MCA diagnoses for the most prevalent system-specific subgroups, accounting for 77% of all MCAs) between THIN and the UK registers of the EUROCAT network has shown that THIN is a valid and complete source of data to investigate MCAs in liveborn children.^40^ We excluded 284 children with records of genetic anomalies or anomalies attributed to known teratogens (e.g. Read codes for anomalies arising from maternal infections or fetal alcohol syndrome).

In addition to system-specific subgroups, we assessed specific types of major heart anomaly to enable the direct comparison of our study with previous literature and grouped the heart anomalies as follows (code lists available from authors): septal defects [including atrial septal defect (ASD), ventricular septal defect (VSD), and atrioventricular septal defect (AVSD)], right ventricular outflow tract defects (RVOTDs), left ventricular outflow tract defects (LVOTDs), and others (including transposition off great vessels, total anomalous pulmonary venous connection, coarctation of the aorta, Ebstein's anomaly, tricuspid atresia and stenosis, patent ductus arterosis (PDA), single ventricle, tetralogy of Fallot, and truncus arteriosus).^27^

### Defining maternal exposure

Clinically recognised maternal depression was defined as diagnoses of depression during the year before conception or in the first trimester. Antenatal exposure to SSRIs and TCAs during the first trimester of pregnancy was defined according to the presence or absence of relevant drug prescriptions in women's records from 4 weeks before to 12 weeks after the first day of the estimated last menstrual period. Using 4 weeks before the last menstrual period enabled the inclusion of drug prescriptions received immediately before pregnancy and potentially used during early pregnancy. We then grouped children into five mutually exclusive exposure groups: no clinical records of maternal depression; maternal depression in the year before conception to the end of the first trimester, but with no antidepressants in the first trimester (unmedicated depression); first-trimester exposure to SSRIs alone; first-trimester exposure to TCAs alone; and dual exposure to both SSRIs and TCAs in the first trimester.

### Statistical analyses

To estimate the disease burden of MCAs overall, and each system-specific subgroup, we calculated absolute risks (per 10 000 live births) for the whole study population and for children in each of the five defined antenatal exposure groups. As SSRIs were the most commonly prescribed antidepressants in the study population, absolute risks were estimated for women prescribed each individual SSRI drug alone (fluoxetine, citalopram, paroxetine, sertraline, and escitalopram), apart from fluvoxamine, which was only prescribed exclusively to 22 women. Children born to women prescribed more than one type of SSRI (236 women) were excluded from the analyses for individual SSRIs.

Logistic regression was used to estimate odds ratios (ORs) with 95% confidence intervals (95% CIs) for MCA overall, and for each system-specific subgroup associated with unmedicated maternal depression, each class of antidepressants, and individual SSRIs during the first trimester of pregnancy. We primarily used children whose mothers did not have clinically recognised depression as the baseline group for all comparisons, so that our results were comparable with the published literature, so that the size of the risk estimates for diagnosed unmedicated depression could be directly compared with those for antidepressant drug exposures, and to maximise the statistical power. Secondly, to assess whether antidepressants were associated with an excess risk of MCA compared with unmedicated depression, we conducted an analysis using children whose mothers had unmedicated depression as the baseline group to estimate ORs for MCAs overall, and the three most common system-specific groups, heart, limb, and genital anomalies, associated with each class of antidepressant and individual SSRIs during the first trimester of pregnancy.

Multivariable analyses were used to adjust all models for maternal characteristics that had been prospectively recorded in women's records before delivery. These were: maternal age; whether women had smoked before or during pregnancy; body mass index (BMI, kg/m^2^), recorded before pregnancy; and socio-economic deprivation, measured using quintiles of the Townsend Index of Deprivation.^41^ As depression is often comorbid with other chronic medical conditions that could be associated with increased risks of congenital anomalies in offspring, we also adjusted for maternal diabetes, hypertension, asthma, and epilepsy in the year before conception or during pregnancy. Missing values for co-variables were fitted as a separate category in the analyses. The generalised estimating equation approach with exchangeable correlation structure was applied to take account of potential clustering between children born to the same woman in consecutive pregnancies.

Studies that compare risk across several drug types in one population can result in spurious associations because of the high number of multiple comparison tests, and this is commonly amplified in congenital anomaly research because of the need to assess the specificity of risk with individual anomalies. Although we recognise statistically significant *P* values may therefore result by chance alone, previous published studies on antidepressant teratogenicity from other countries used 95% CIs,^14^,^16^,^22^,^24^,^25^,^27^ so we did not carry out specific adjustment for multiple comparisons to ensure our findings could be directly comparable. Furthermore, statistical adjustment for multiple comparison tests assume that there is one overall null hypothesis, and thus one research question, whereas our aim was to assess multiple drugs across several types of anatomical groups, as a key objective was to assess whether there were different specific drug–anomaly associations. Multiple comparisons are thus often inevitable in congenital anomaly research; however, for all adjusted ORs we described the exact numbers of exposed cases available, and for associations where 95% CIs did not cross 1.00, we additionally presented exact *P* values to three decimal places in consideration that we would expect smaller values of *P *< 0.01 to be less likely as a result of chance alone.

All analyses were carried out using stata SE 11.0 (Stata Corp., College Station, TX, USA).

## Results

Among 349 127 liveborn singletons, the overall prevalence of MCA was 2.7% (95% CI 2.6–2.8%). For children with MCA, their mothers had a similar sociodemographic profile to mothers of children without MCA (Table1); however, higher proportions of mothers of children with MCA had chronic medical comorbidities, particularly diabetes and epilepsy, than those of children with no MCA. Of all children, 3.8% had mothers with depression that was not treated with antidepressant medication during the first trimester (diagnosed but unmedicated depression), whereas 2.2 and 0.7% had mothers with first-trimester exposure to SSRIs alone and TCAs alone, respectively. The most commonly prescribed SSRIs during the first trimester of pregnancy were fluoxetine (0.9%), citalopram (0.6%), and paroxetine (0.3%). Maternal characteristics for pregnancies in women with unmedicated depression or antidepressant use showed higher socio-economic deprivation, smoking, obesity, and asthma, compared with pregnancies in women with no depression (Table S1). In particular, women with medicated depression were slightly more likely to have pre-existing diabetes, hypertension, and epilepsy than women with unmedicated depression; however, distributions were similar across antidepressant classes and individual SSRIs (Tables S1 and S2).

**Table 1:** Maternal characteristics for singletons born with and without major congenital anomalies

	All children		Children without MCAs		Children with MCAs	
	*n* =* *349 127		*n *=* *339 730		*n *=* *9397	
	*n*	%	*n*	%	*n*	%
**Maternal age at the end of pregnancy, years (Median [interquartile range])**	30 (26–34)		30 (26–34)		30 (26–34)	
**Townsend deprivation index**						
1 (least deprived)	85 160	24.4	82 850	24.4	2310	24.6
2	67 968	19.5	66 193	19.5	1775	18.9
3	68 224	19.5	66 368	19.5	1856	19.8
4	63 284	18.1	61 596	18.1	1688	18.0
5 (most deprived)	47 190	13.5	45 850	13.5	1340	14.3
Missing	17 301	5.0	16 873	5.0	428	4.6
**Ever smoked before delivery**	132 934	38.1	129 415	38.1	3519	37.4
**BMI before pregnancy (kg/m**^**2**^**)**						
Underweight (<18.5)	11 335	3.2	11 026	3.2	309	3.3
Normal (18.5–24.9)	154 140	44.2	150 128	44.2	4012	42.7
Overweight (25–29.9)	58 998	16.9	57 407	16.9	1591	16.9
Obese (30–39.9)	32 130	9.2	31 154	9.2	976	10.4
Missing	92 524	26.5	90 015	26.5	2509	26.7
**Diabetes**	1619	0.5	1521	0.4	98	1.0
**Hypertension**	919	0.3	877	0.3	42	0.4
**Asthma**	26 981	7.7	26 204	7.7	777	8.3
**Epilepsy**	1431	0.4	1353	0.4	78	0.8

Table2 shows the numbers and absolute risks of any MCA and system-specific anomalies for children with different antenatal exposures, and Table3 shows adjusted ORs (aORs). Children born to women with diagnosed depression unmedicated in early pregnancy had higher absolute risks of MCAs than children of mothers with no depression (283/10 000 and 268/10 000, respectively; Table2), although the relative risk was not statistically significant (aOR 1.07, 95% CI 0.96–1.18; Table3). Similarly, there were no increased risks of MCA overall associated with SSRIs, TCAs, or joint exposure.

**Table 2:** Absolute risks (per 10 000 live births) of major congenital anomalies in children according to first-trimester exposure to unmedicated depression and antidepressant medications

	All children		No depression		Depression*		SSRIs alone		TCAs alone		SSRIs & TCAs**	
	*n *=* *349 127		*n *=* *325 294		*n *=* *13 432		*n *=* *7683		*n *=* *2428		*n *=* *290	
	*n*	*n*/10 000	*n*	*n*/10 000	*n*	*n*/10 000	*n*	*n*/10 000	*n*	*n*/10 000	*n*	*n*/10 000
**All MCAs combined**	9397	269	8731	268	380	283	204	266	74	305	8	276
Heart	2646	76	2444	75	112	83	68	89	20	82	2	69
Limb	1868	54	1750	54	71	53	33	43	14	58	0	0
Genital system	1391	40	1292	40	67	50	22	29	8	33	2	69
Urinary system	886	25	815	25	37	28	23	30	9	37	2	69
Chromosomal	592	17	551	17	27	20	10	13	4	16	0	0
Orofacial cleft	472	14	438	13	21	16	11	14	2	8	0	0
Nervous system	513	15	465	14	24	18	15	20	7	29	2	69
Musculoskeletal system	468	13	442	14	14	10	9	12	2	8	1	34
Digestive system	338	10	313	10	11	8	11	14	3	12	0	0
Eye	332	10	313	10	9	7	6	8	4	16	0	0
Other anomalies***	328	9	305	9	14	10	7	9	2	8	0	0
Respiratory system	222	6	205	6	5	4	8	10	4	16	0	0
Ear, face, and neck	90	3	83	3	7	5	0	0	0	0	0	0
Abdominal wall	74	2	69	2	2	1	3	4	0	0	0	0

* Diagnosed in the mother in the year before conception up to the end of the first trimester, but with no antidepressant drug prescriptions in the first trimester.

** Dual exposure to both drug classes in the first trimester of pregnancy.

*** For example, asplenia, situs inversus, and skin disorders.

**Table 3:** Adjusted odds ratios for major congenital anomalies in children with first-trimester exposure to unmedicated maternal depression and antidepressant medications (*n *=* *349 127 children, 9397 with major congenital anomalies)

	Depression*		SSRIs alone		TCAs alone		SSRIs & TCAs**	
	*n *=* *13 432		*n *=* *7683		*n *=* *2428		*n *=* *290	
	aOR***	95% CI	aOR***	95% CI	aOR***	95% CI	aOR***	95% CI
**All MCAs combined**	1.07	0.96–1.18	1.01	0.88–1.17	1.09	0.87–1.38	1.02	0.50–2.06
Heart	1.10	0.91–1.33	1.14	0.89–1.45	1.03	0.65–1.63	0.85	0.21–3.42
Limb	1.03	0.81–1.31	0.88	0.62–1.25	1.04	0.61–1.77	–	
Genital system	1.25	0.98–1.60	0.71	0.46–1.08	0.81	0.41–1.63	1.67	0.41–6.79
Urinary system	1.10	0.79–1.54	1.20	0.79–1.82	1.46	0.76–2.83	2.75	0.68–11.05
Chromosomal	1.29	0.87–1.93	0.79	0.42–1.48	0.90	0.34–2.43	–	
Orofacial cleft	1.13	0.73–1.75	1.06	0.58–1.93	0.58	0.14–2.31	–	
Nervous system	1.23	0.81–1.86	1.39	0.82–2.34	1.86	0.88–3.92	4.57	1.10–19.06*****
Musculoskeletal system	0.78	0.46–1.33	0.91	0.44–1.88	0.56	0.14–2.33	2.66	0.39–18.10
Digestive system	0.84	0.46–1.54	1.43	0.79–2.61	1.26	0.40–3.94	–	
Eye	0.70	0.36–1.38	0.82	0.36–1.86	1.68	0.63–4.50	–	
Other anomalies****	1.21	0.69–2.13	1.30	0.61–2.77	0.83	0.19–3.58	–	
Respiratory system	0.57	0.23–1.38	1.56	0.77–3.15	2.50	0.93–6.69	–	
Ear, face, and neck	2.39	1.08–5.27******	–		–		–	
Abdominal wall	0.53	0.13–2.17	1.41	0.43–4.59	–		–	

* Diagnosed in the mother in the year before conception up to the end of the first trimester, but with no antidepressant drug prescriptions.

** Dual exposure to both drug classes in the first trimester of pregnancy.

*** Odds ratio compared with children born to mothers without clinically recognised depression, adjusted for maternal age at the end of pregnancy, year of childbirth, Townsend deprivation quintile, maternal smoking history, body mass index before pregnancy, and maternal diabetes, hypertension, asthma, and epilepsy in the year before conception or during pregnancy.

**** For example, asplenia, situs inversus, and skin disorders.

***** *P *=* *0.037.

****** *P *=* *0.031.

With few exceptions, the absolute risks of system-specific congenital anomalies were similar for children across the different antenatal exposure groups, showing no substantial increases compared with children born to women without depression. Absolute risks of heart anomalies were higher in children born to women with unmedicated depression and in children exposed to SSRIs or TCAs in early pregnancy, compared with children of mothers with no depression (Table2); however, the aORs ranged between 1.03 and 1.14, and none were statistically significant (Table3). Similarly, results for other system-specific anomaly groups were not statistically significant, apart from a two-fold increased risk of ear, face, and neck anomalies in children born to women with diagnosed depression unmedicated in the first trimester (aOR 2.39, 95% CI 1.08–5.27, *P *= 0.031) and an increase of nervous system anomalies in children with joint SSRI and TCA exposure in the first trimester (aOR 4.57, 95% CI 1.10–19.06, *P *= 0.037; Table3). When examining specific types of heart anomaly, there were again no statistically significant increased risks for children born to women with unmedicated depression in the first trimester or for children exposed to SSRIs or TCAs, compared with children of women with no depression (Table4).

**Table 4:** Absolute risks (per 10 000 live births) and adjusted odds ratios for specific heart anomalies in children with first-trimester exposure to unmedicated maternal depression and antidepressant medications (*n *=* *349 127 children, 2646 with heart anomalies)

MCAs	No depression	Depression*		SSRIs alone		TCAs alone	
	*n *=* *325 294	*n *=* *13 432		*n *=* *7683		*n *=* *2428	
	*n*/10 000	*n*/10 000	aOR (95% CI)**	*n*/10 000	aOR (95% CI)**	*n*/10 000	aOR (95% CI)**
**Heart**							
Septal defect***	47	51	1.09 (0.86–1.39)	43	0.89 (0.63–1.27)	49	0.98 (0.55–1.73)
ASD	10	9	0.85 (0.48–1.51)	18	1.68 (0.98–2.91)	16	1.43 (0.54–3.83)
VSD	33	36	1.09 (0.81–1.45)	21	0.63 (0.38–1.03)	33	0.93 (0.46–1.87)
RVOTD	3	5	1.58 (0.73–3.40)	8	2.22 (0.98–5.03)	4	1.19 (0.17–8.23)
LVOTD	1	1	1.59 (0.36–7.16)	1	1.50 (0.20–11.24)	4	4.72 (0.63–35.25)
Other****	33	40	1.20 (0.90–1.58)	44	1.27 (0.90–1.80)	33	0.94 (0.43–2.04)

* Diagnosed in the mother in the year before conception up to the end of the first trimester, but with no antidepressant drug prescriptions.

** Odds ratio compared with children born to mothers without clinically recognised depression, adjusted for maternal age at the end of pregnancy, year of childbirth, Townsend deprivation quintile, maternal smoking history, body mass index before pregnancy, and maternal diabetes, hypertension, asthma, and epilepsy in the year before conception or during pregnancy.

*** Atrial, ventricular, or combined septal defects.

**** Transposition of great vessels, total anomalous pulmonary venous connection, coarctation of the aorta, Ebstein's anomaly, tricuspid atresia and stenosis, patent ductus arterosis, single ventricle, tetralogy of Fallot, truncus arteriosus.

Compared with an absolute risk of 268/10 000 children whose mothers had no depression and 283/10 000 children whose mothers had unmedicated depression, absolute risks of overall MCAs were lower for children exposed to fluoxetine, citalopram, or escitalopram in early pregnancy (241, 267 and 210/10 000, respectively), but higher for children exposed to paroxetine or sertraline (300 and 330/10 000, respectively) (Table5). For heart anomalies, compared with absolute risks of 75/10 000 children whose mothers had no depression and 83/10 000 children whose mothers had unmedicated depression, paroxetine and sertraline had the highest absolute risks (142 and 119/10 000, respectively), followed by escitalopram (90/10 000) and citalopram (87/10 000), whereas fluoxetine showed a lower risk (66/10 000). There was wide variation in adjusted odds ratios for MCA overall and for system-specific subgroups associated with each SSRI drug: some were above and others below 1.00, and many 95% CIs were wide (Table5). A 78% increase in congenital heart anomalies was found among children with paroxetine exposure alone (aOR 1.78, 95% CI 1.09–2.88, *P *= 0.020). This was based on 17 affected children among the 1200 exposed to paroxetine who had a range of heart anomalies [eight with septal defects (three with ASD, four with VSD, one with AVSD), one with ASD and PDA, four with PDA, one with RVOTD (pulmonary infundibular stenosis), one with transposition of the great vessels, and two with unspecified heart anomalies]. Despite the higher absolute risks of overall MCA and heart anomalies in children of mothers prescribed with sertraline, the aORs were not statistically significant (aOR 1.27, 95% CI 0.85–1.89; aOR 1.52, 95% CI 0.78–2.96, respectively). There were also statistically significant increased risks of urinary and digestive system anomalies associated with citalopram exposure (*P *= 0.025 and 0.035, respectively), based on ten and five cases, respectively, among the 1946 children with citalopram exposure (Table5). We also found an increased risk of respiratory system anomalies associated with sertraline (aOR 4.04, 95% CI 1.00–16.27, *P *= 0.049), based on five exposed cases, and a decreased risk of genital system anomalies with fluoxetine exposure (aOR 0.38, 95% CI 0.16–0.93, *P *= 0.034), based on five exposed cases (Table5).

**Table 5:** Absolute risks (per 10 000 live births) and adjusted odds ratios for major congenital anomalies in children born to women exclusively prescribed specific SSRIs during the first trimester of pregnancy

	SSRIs during the first trimester of pregnancy*									
	Fluoxetine		Citalopram		Paroxetine		Sertraline		Escitalopram	
	*n *=* *3189		*n *=* *1946		*n *=* *1200		*n *=* *757		*n *=* *333	
	*n*/10 000	aOR (95% CI)**	*n*/10 000	aOR (95% CI)**	*n*/10 000	aOR (95% CI)**	*n*/10 000	aOR (95% CI)**	*n*/10 000	aOR (95% CI)**
**All MCAs combined**	241	0.91 (0.73–1.15)	267	1.06 (0.80–1.40)	300	1.08 (0.77–1.50)	330	1.27 (0.85–1.89)	210	0.85 (0.40–1.81)
Heart	66	0.84 (0.55–1.30)	87	1.13 (0.70–1.82)	142	1.78 (1.09–2.88)****	119	1.52 (0.78–2.96)	90	1.15 (0.36–3.65)
Limb	44	0.89 (0.52–1.50)	31	0.68 (0.30–1.53)	50	0.92 (0.41–2.06)	66	1.36 (0.57–3.28)	30	0.69 (0.10–4.89)
Genital system	16	0.38 (0.16–0.93)*****	36	0.91 (0.43–1.93)	42	0.97 (0.40–2.37)	13	0.32 (0.04–2.40)	30	0.80 (0.11–5.78)
Urinary system	28	1.14 (0.59–2.19)	51	2.07 (1.10–3.92)******	25	0.99 (0.32–3.10)	13	0.54 (0.08–3.76)	0	–
Chromosomal	6	0.38 (0.09–1.15)	5	0.34 (0.05–2.37)	17	0.91 (0.22–3.83)	40	2.32 (0.74–7.27)	0	–
Orofacial cleft	22	1.64 (0.78–3.45)	15	1.14 (0.36–3.60)	0	–	13	0.99 (0.14–6.96)	0	–
Nervous system	25	1.77 (0.87–3.57)	15	1.18 (0.37–3.71)	0	–	26	1.79 (0.42–7.54)	30	2.45 (0.34–17.48)
Musculoskeletal system	13	1.01 (0.38–2.65)	10	0.81 (0.11–5.99)	8	0.56 (0.07–4.53)	26	2.13 (0.51–8.90)	0	–
Digestive system	13	1.26 (0.47–3.39)	26	2.60 (1.07–6.32)*******	0	–	26	2.69 (0.67–10.76)	0	–
Eye	9	0.96 (0.30–3.05)	5	0.55 (0.08–3.95)	8	0.83 (0.12–5.76)	0	–	30	3.23 (0.41–25.26)
Other anomalies***	9	1.26 (0.39–4.02)	10	1.77 (0.43–7.18)	0	–	13	1.84 (0.27–12.79)	0	–
Respiratorysystem	13	1.90 (0.71–5.09)	5	0.79 (0.11–5.69)	8	1.25 (0.18–8.96)	26	4.04 (1.00–16.27)********	0	–
Ear, face, and neck	0	–	0	–	0	–	0	–	0	–
Abdominal wall	3	1.14 (0.16–8.19)	0	–	0	–	13	4.90 (0.67–36.01)	0	–

* Children born to women treated with each specific SSRI drug exclusively during the first trimester, excluding children born to women treated with fluvoxamine (22 women) or with more than one type of SSRI (236 women: 71% were co-prescribed with fluoxetine).

** Odds ratio compared with children born to mothers without clinically recognised depression, adjusted for maternal age at the end of pregnancy, year of childbirth, Townsend deprivation index, maternal smoking history, maternal body mass index before pregnancy, and maternal diabetes, hypertension, asthma, and epilepsy in the year before conception or during pregnancy.

*** For example, asplenia, situs inversus, and skin disorders.

**** *P *=* *0.020.

***** *P *=* *0.034.

****** *P *=* *0.025.

******* *P *=* *0.035.

******** *P *=* *0.049.

When we used women with diagnosed but unmedicated depression as the baseline group (Table6), most point estimates of aORs associated with SSRIs, TCAs, and individual SSRI drugs decreased slightly. No statistically significant associations of overall and system-specific congenital anomaly risks were found, apart from a decreased risk of genital system anomaly with SSRI exposure, and specifically fluoxetine exposure (aOR 0.31, 95% CI 0.12–0.76, *P *= 0.024); however, slightly higher point estimates of congenital anomaly risk in children exposed to paroxetine and sertraline remained in comparison with children of mothers with unmedicated depression (Table6). The aORs for congenital heart anomalies in children exposed to paroxetine and sertraline were 1.67 (95% CI 1.00–2.80, *P *= 0.051) and 1.39 (95% CI 0.70–2.74, *P *= 0.345), respectively, which were similar to the previous estimates compared with children born to women without diagnosed depression (Tables5 and 6).

**Table 6:** Adjusted odds ratios for major congenital anomalies in children with first-trimester exposure to different antidepressant medications, compared with children of women with unmedicated depression (*n *=* *23 833 children, 666 with major congenital anomalies)

	SSRIs alone		TCAs alone		SSRIs & TCAs*		Fluoxetine alone		Citalopram alone		Paroxetine alone		Sertraline alone		Escitalopram alone	
	*n *=* *7683		*n *=* *2428		*n *=* *290		*n *=* *3189		*n *=* *1946		*n *=* *1200		*n *=* *757		*n *=* *333	
	aOR**	95% CI	aOR**	95% CI	aOR**	95% CI	aOR**	95% CI	aOR**	95% CI	aOR**	95% CI	aOR**	95% CI	aOR**	95% CI
**All MCAs**	0.93	0.78–1.11	1.02	0.79–1.32	0.94	0.46–1.92	0.85	0.66–1.09	0.97	0.71–1.31	1.01	0.71–1.44	1.17	0.78–1.77	0.77	0.36–1.66
Heart	1.04	0.76–1.41	0.90	0.54–1.50	0.78	0.19–3.27	0.79	0.49–1.26	1.02	0.61–1.70	1.67	1.00–2.80***	1.39	0.70–2.74	1.09	0.34–3.50
Limb	0.82	0.54–1.25	1.08	0.60–1.93	–		0.83	0.47–1.49	0.59	0.26–1.37	0.94	0.41–2.15	1.29	0.52–3.20	0.58	0.08–4.11
Genital system	0.57	0.35–0.92****	0.62	0.29–1.30	1.35	0.33–5.50	0.31	0.12–0.76	0.71	0.32–1.56	0.81	0.32–2.02	0.26	0.04–1.87	0.57	0.08–4.17

* Dual exposure to both drug classes in the first trimester of pregnancy.

** Odds ratio compared with children born to mothers with depression unmedicated with antidepressants in the first trimester, adjusted for maternal age at the end of pregnancy, year of childbirth, Townsend deprivation quintile, maternal smoking history, body mass index before pregnancy, and maternal diabetes, hypertension, asthma, and epilepsy in the year before conception or during pregnancy.

*** *P *=* *0.051.

**** *P *=* *0.024.

## Discussion

### Main findings

Overall, MCA and system-specific anomaly risks were similar in children of mothers with and without antidepressants (SSRIs or TCAs) in early pregnancy. For individual SSRIs, we found no evidence of teratogenicity for most antidepressants, except for an increase in heart anomalies associated with paroxetine and an excess risk of similar magnitude (though not statistically significant) with sertraline. Most heart anomalies in children exposed to paroxetine were relatively mild conditions, with no evidence that exposure was related to a specific type of heart anomaly. Citalopram was associated with increased urinary and digestive anomalies; however, these were based on fewer exposed cases.

### Strengths and limitations

Our study is among few that have examined the relative safety across antidepressant classes, and is the first UK study to assess individual SSRIs with system-specific anomalies in a single population. With over 2400 TCA exposures, 7600 SSRI exposures, and over 13 000 mothers with diagnosed unmedicated depression, the statistical power was greater or similar to previous population-based studies from Europe and North America.^22^,^24^–^27^,^29^–^31^ Power was inevitably reduced when examining system-specific groups, and given the number of comparisons conducted, we cannot rule out random error, which is also a limitation of previous studies.

We included MCAs diagnosed up to age 20 years, where available, so we expect to have captured these for live births as completely if not more completely than registry data.^40^ As stillborn children are not registered with a general practice (GP) we only included liveborn children, as in most previous studies. Stillbirth occurs in approximately 0.6% of births in developed countries,^42^ and congenital anomalies account for only 8–14% of stillbirths,^43^,^44^ so the effect of excluding them on our estimates should be minimal. We were also unable to ascertain MCAs among pregnancies ending in spontaneous or induced abortions, which may underestimate teratogenicity, a limitation that no studies have overcome. There are no accurate data sources to ascertain congenital anomalies in pregnancies than end in spontaneous abortion because many occur early in gestation when women may not know they are pregnant, and autopsy information on later losses is rarely ascertained. This is similar for induced abortions, as most are carried out early in pregnancy. UK registry ascertainment of MCAs among medically terminated pregnancies varies regionally,^45^ and national abortion statistics estimate <1% are because of an MCA.^46^ In the same primary care population used in this study, we found women exposed to SSRIs or TCAs had increased risks of both spontaneous and induced abortion; women continuing SSRI or TCA treatment when pregnant had higher risks than women who discontinued, particularly those on SSRIs.^35^ A lack of information on the reasons for these spontaneous and induced abortions, and potential confounding by changes in severity of the underlying depression, limit our ability to determine whether these were teratogenic effects.

The prevalence of diagnosed maternal depression in UK primary care is similar to estimates from surveys using clinical diagnostic criteria.^47^ This enabled us to assess effects of underlying maternal depression on MCA risks in offspring, and to compare risks between women with medicated and unmedicated depression, which has not been achieved previously. Although the effects of psychotropic drugs can never be completely separated from more severe illness itself, such estimates are less likely to be influenced by residual confounding effects, as when comparing with women not suffering from depression. Moreover, as all pregnant women in the UK must be registered with a GP to benefit from free antenatal care and prescriptions, it is unlikely that women with psychotropic drug prescriptions were not identified. Women receiving prescriptions may not have actually taken the medication during the organogenetic period, which could bias estimates towards a null effect; however, studies large enough to assess congenital anomaly risks are limited in their ability to obtain data on actual medication consumption.

Exposure, outcome, and covariate data were prospectively recorded, thereby minimising recall bias. Maternal sociodemographics and comorbidities were included in the statistical models to minimise confounding; however, misclassification of some covariables was possible if not recorded in a timely manner in the GP records.

### Interpretation

To our knowledge, no studies have assessed congenital anomaly risks in women with clinically diagnosed depression who were not prescribed antidepressants in early pregnancy. A Danish study found an increased heart anomaly risk in children of 806 women treated with SSRIs immediately before and after, but not during, pregnancy, which was similar to the increased risk in 4183 women exposed to SSRIs in pregnancy.^29^ Confounding by indication may therefore contribute to drug-associated risks, but 806 women (0.1% of the overall population of 848 786 women) would not have accounted for the majority of women with unmedicated depression.

Five studies have examined the comparative safety of SSRIs and TCAs in a single population.^12^,^25^,^29^,^30^,^36^ A UK GP data study of approximately 3000 pregnancies exposed to antidepressants assessed only class-level effects, and found no MCA risks; however, their inclusion of stillbirths and terminations probably distorted the true risks, as congenital anomalies are rarely recorded for these pregnancies in GP data.^30^ Two Swedish studies have found that heart anomalies were associated with paroxetine exposure (OR 1.66, 95% CI 1.09–2.53, based on 24 exposed cases), but not with SSRIs overall, which is consistent with our findings.^25^,^36^ They also found a similar TCA-associated risk (OR 1.63, 95% CI 1.12–2.36).^25^ In a USA study, limb anomalies and spina bifida were associated with TCAs.^12^ Although we did not find overall MCA increases with TCAs, we found an increase of nervous system anomalies in children with dual TCA and SSRI exposure. These risks (including the one in our study) were based on between one and eight exposed cases only, and have not been consistently reported in other studies.

A US case–control study including more than 300 MCA cases with first-trimester SSRI exposure found increases of RVOTD associated with paroxetine (OR 3.3, 95% 1.3–8.8), and increases of septal defect associated with sertraline (OR 2.0, 95% CI 1.2–4.0).^14^ Similar increases of heart anomalies with paroxetine and sertraline were found in other studies from the USA, Denmark, and Finland.^16^,^22^,^24^,^27^ The consistency of a modest increase of heart anomalies with paroxetine and sertraline is reassuring for the validity of our results. Results for other individual SSRIs are inconsistent. The Finish study also reported associations between RVOTD and fluoxetine (OR 2.0, 95% CI 1.3–3.2) and between neural tube defects and citalopram (OR 2.5, 95% CI 1.2–5.1).^27^ In contrast, the Danish study reported a nearly three-fold increase of septal defect with citalopram (OR 2.5, 95% 1.0–6.1), but not with fluoxetine (OR 1.3, 95% CI 95% 0.3–5.4),^22^ and later re-analysis showed small increases for some heart anomalies for all individual SSRIs, apart from escitalopram.^29^ As heart anomalies are by far the most common MCA, the power to detect risks for other system-specific groups is limited across all published studies. Nevertheless, as drug-specific effects for non-cardiovascular anomalies have been largely inconsistent in the existing literature, there is no strong evidence that any individual SSRIs increase their risk.

## Conclusion

Children whose mothers were prescribed SSRIs or tricyclic antidepressants (TCAs) in early pregnancy were not at an increased risk of major congenital anomaly overall, nor were children whose mothers had diagnosed but unmedicated depression. We provide absolute and relative major congenital anomaly (MCA) risks for individual antidepressants to facilitate health professionals' discussions of the potential risks and benefits of treated and untreated depression with pregnant women, or with those planning to conceive. For example, where approximately eight children out of 1000 born to women without depression are expected to have congenital heart anomalies, approximately seven additional cases could occur if children were exposed to paroxetine.

Children born to women with medicated and unmedicated depression have, overall, similar risks of having MCAs as those born to women without depression. Our findings, together with findings from other studies, however, indicate that children born to women prescribed paroxetine have a small excess risk of congenital heart anomalies, although most have relatively mild conditions. Although this may indicate real teratogenicity, most studies were conducted after the specific pharmaceutical warning against paroxetine in 2005,^7^ so increased international concerns of its potential risks may have led to increased monitoring and early diagnosis in children exposed to antidepressants, particularly SSRIs. Evidence to disfavour certain SSRIs regarding teratogenicity remains weak, and all seem largely safe in terms of MCA risks.

### Disclosure of interests

L.S. received grants from the Wellcome Trust and personal fees from GlaxoSmithKline. All other authors report no competing interests.

### Contribution to authorship

L.B., J.E.G., J.W., L.F., R.S., L.S., P.D., R.B.H., and L.J.T. contributed to the conception and design of the study, and to the analysis and interpretation of the study results. L.B. and L.J.T. analysed the data. L.B. and L.J.T. drafted the article. L.B., J.E.G., J.W., L.F., R.S., L.S., P.D., R.B.H., and L.J.T. revised the article and gave approval of the final version. L.J.T. supervised the study and is guarantor.

### Details of ethics approval

All data are anonymised, such that individual patients as well as the name and specific location of general practices cannot be identified by researchers. Ethical approval for this study was obtained from the Medical Research Ethics Committee, administered and approved by the National Health Service South East Research Ethics Committee (REC reference 04/MRE01/9).

### Funding

This work was funded by a grant from the Wellcome Trust. The researchers conducted this study independent of the funder.

## Supporting Information

Table S1 Maternal characteristics for singleton pregnancies in women with different antenatal exposures. **Table S2.** Maternal characteristics for singletons with different SSRI exposures in the first trimester of pregnancy.
