# Supplementary material for: Maternal depression, antidepressant prescriptions, and congenital anomaly risk in offspring: a population-based cohort study
Source: BJOG. 2014 Mar 11;121(12):1471–81. doi: 10.1111/1471-0528.12682 (PMC4232879; doi:10.1111/1471-0528.12682)
Supplement: Table S1 — Maternal characteristics for singleton pregnancies in women with different antenatal exposures. Table S2. Maternal characteristics for singletons with different SSRI exposures in the first trimester of pregnancy. [file bjo0121-1471-sd1.pdf]

Table S1: Maternal characteristic for singleton pregnancies in women with different antenatal exposures

	No depression		Depression[a]		SSRIs alone		TCAs alone		SSRIs & TCAs[b]	
	N=325,294		n=13,432		n=7,683		n=2,428		n=290	
	n	%	n	%	n	%	n	%	n	%
**Maternal age** at the end of pregnancy,years Median (IQR)	30 (26-34)		29 (24-33)		30 (25-34)		30 (26-34)		30 (25-34)	
**Townsend deprivation index**										
1 (Least deprived)	81,101	24.9	2,292	17.1	1,292	16.8	423	17.4	52	17.9
2	64,131	19.7	2,200	16.4	1,198	15.6	399	16.4	40	13.8
3	63,510	19.5	2,663	19.8	1,558	20.3	433	17.8	60	20.7
4	57,953	17.8	3,031	22.6	1,699	22.1	538	22.2	63	21.7
5 (Most deprived)	42,511	13.1	2,618	19.5	1,483	19.3	524	21.6	54	18.6
Missing	16,088	4.9	628	4.7	453	5.9	111	4.6	21	7.2
**Ever smoked before delivery**	119,679	36.8	7,246	53.9	4,587	59.7	1,239	51.0	183	63.1
**BMI** before pregnancy (kg/m^2)										
Under-weight (<18.5)	10,345	3.2	555	4.1	321	4.2	101	4.2	13	4.5
Normal (18.5-24.9)	144,070	44.3	5,704	42.5	3,211	41.8	1,055	43.5	100	34.5
Over-weight(25-29.9)	54,393	16.7	2,592	19.3	1,506	19.6	451	18.6	56	19.3
Obese (30-39.9)	28,783	8.8	1,781	13.3	1,189	15.5	314	12.9	63	21.7
Missing	87,703	27.0	2,800	20.8	1,456	19.0	507	20.9	58	20.0
**Diabetes**	1,455	0.4	72	0.5	70	0.9	18	0.7	4	1.4
**Hypertension**	818	0.3	40	0.3	41	0.5	19	0.8	1	0.3
**Asthma**	23,945	7.4	1,606	12.0	1,035	13.5	353	14.5	42	14.5
**Epilepsy**	1,288	0.4	68	0.5	48	0.6	23	0.9	4	1.4

[a] diagnosed in the mother in the year before conception up to the end of the first trimester, but with no antidepressant drug prescriptions in the first trimester
[b] dual exposure to both drug classes in the first trimester of pregnancy
IQR=interquartile range
BMI=body mass index

Table S2: Maternal characteristic for singletons with different SSRI exposures in the first trimester of pregnancy[a]

	Fluoxetine		Citalopram		Paroxetine		Sertraline		Escitalopram	
	n=3,189		n=1,946		n=1,200		n=757		n=333	
	n	%	n	%	n	%	n	%	n	%
**Maternal age** at the end of pregnancy,years Median (IQR)	30 (25-34)		29 (25-34)		30 (26-34)		30 (25-34)		29 (25-35)	
**Townsend deprivation index**										
1 (Least deprived)	546	17.1	328	16.9	222	18.5	114	15.1	50	15.0
2	482	15.1	298	15.3	205	17.1	124	16.4	44	13.2
3	664	20.8	398	20.5	237	19.8	151	19.9	64	19.2
4	699	21.9	457	23.5	254	21.2	160	21.1	68	20.4
5 (Most deprived)	601	18.8	371	19.1	217	18.1	166	21.9	75	22.5
Missing	197	6.2	94	4.8	65	5.4	42	5.5	32	9.6
**Ever smoked before delivery**	1,873	58.7	1,252	64.3	638	53.2	438	57.9	216	64.9
**BMI** before pregnancy (kg/m^2)										
Under-weight (<18.5)	133	4.2	309	4.2	57	4.8	23	3.0	13	3.9
Normal (18.5-24.9)	1,330	41.7	3,102	41.8	526	43.8	320	42.3	136	40.8
Over-weight(25-29.9)	637	20.0	1,459	19.6	225	18.8	147	19.4	67	20.1
Obese (30-39.9)	489	15.3	1,147	15.4	156	13.0	120	15.9	60	18.0
Missing	600	18.8	1,408	19.0	236	19.7	147	19.4	57	17.1
**Diabetes**	29	0.9	70	0.9	70	0.9	70	0.9	70	0.9
**Hypertension**	11	0.3	18	0.9	4	0.3	6	0.8	2	0.6
**Asthma**	418	13.1	285	14.6	145	12.1	103	13.6	53	15.9
**Epilepsy**	20	0.6	13	0.7	7	0.6	5	0.7	1	0.3

[a] Children born to women treated with each specific SSRI drug exclusively during the first trimester, excluding children born to women treated with fluvoxamine (22 women) or with more than one type of SSRI (236 women: 71% co-prescribed with fluoxetine)
IQR=interquartile range
BMI=body mass index
